# Epidemiology of Peripheral Artery Disease: Narrative Review

**DOI:** 10.3390/life12071041

**Published:** 2022-07-12

**Authors:** Lilla Horváth, Noémi Németh, Gergely Fehér, Zsuzsanna Kívés, Dóra Endrei, Imre Boncz

**Affiliations:** 1 Centre for Occupational Medicine, Medical School, University of Pécs, 7624 Pécs, Hungary; feher.gergely@pte.hu; 2 Doctoral School, Faculty of Health Sciences, University of Pécs, 7621 Pécs, Hungary; noemi.nemeth@etk.pte.hu; 3 Department of Primary Health Care, University of Pécs, 7623 Pécs, Hungary; 4 Institute for Health Insurance, Faculty of Health Sciences, University of Pécs, 7621 Pécs, Hungary; kives.zsuzsanna@etk.pte.hu (Z.K.); endrei.dora@pte.hu (D.E.); imre.boncz@etk.pte.hu (I.B.); 5 1st Department of Internal Medicine, Medical School, University of Pécs, 7624 Pécs, Hungary; 6 National Laboratory for Human Reproduction, University of Pécs, 7624 Pécs, Hungary

**Keywords:** peripheral artery disease (PAD), epidemiology, disease burden, PAD prevalence, amputation

## Abstract

Past decades have witnessed a major epidemiologic transition with a considerable increase in the disease burden associated with atherosclerotic cardiovascular diseases (CVDs), with low-income and middle-income countries (LMICs) experiencing substantial increase in CVDs. As the global population is aging and peripheral artery disease (PAD) is strongly age-related, it is estimated to become increasingly prevalent in the future. PAD shares risk factors with coronary and cerebrovascular risk factors, particularly diabetes mellitus and smoking, and is associated with significant CVD morbidity and mortality. Despite advances in therapeutic modalities, 236 million people were estimated to be suffering from PAD worldwide in 2015, and numbers have been rising since. The prevalence of asymptomatic PAD has remained high; PAD prevalence seems higher among women and is related to ethnicity. Although several epidemiological studies have been published on PAD during the past decades, data from LMICs are scarce. Besides providing up-to-date epidemiological data retrieved from the literature and the Global Burden of Disease (GBD) study database, this narrative review also intends to draw attention to the substantial disease burden of PAD manifesting in more Years of Life Lost (YLL), age-adjusted mortality and amputation rates, with a special focus on some European countries and especially Hungary, i.e., the country with the highest amputation rate in Europe.

## 1. Introduction

Noncommunicable diseases (NCDs) have emerged as leading causes of morbidity and mortality in developed and developing regions of the world. The disease burden attributed to NCDs continues to increase worldwide, with cardiovascular diseases (CVDs) the leading cause of mortality and morbidity. Consequently, the prevalence of peripheral artery disease (PAD), also called lower extremity peripheral artery disease (LEAD), has been rising markedly [1,2,3]. PAD is a progressive atherosclerotic disease of the lower extremities and is considered an indicator of generalised atherosclerosis [4,5,6].

The most common symptom of PAD is claudication, cramping pain in the calves, thighs or buttocks, characteristically triggered by walking and subsiding with rest. Further symptoms may also include atypical pain on exertion and ischaemic pain at rest. The final stages may result in tissue loss and amputation. PAD may remain asymptomatic for a while, but symptomatic PAD is associated with severe limitations in physical function, especially walking and a wide range of daily activities [7,8,9,10]. A large majority of patients remain asymptomatic even with an ABI > 0.9. Asymptomatic Peripheral Artery Disease (APAD) combined with traditional risk factors (hypertension, diabetes mellitus and smoking) substantially elevates cardiovascular (CV) risk. Patients with suspected underlying disease should undergo further noninvasive tests [11,12].

PAD has been shown to be associated with significant morbidity and mortality from cardiovascular disease (CVD), myocardial infarction (MI), stroke and major adverse coronary events (MACE). Patients suffering from PAD have been found to have an equal risk of a subsequent stroke or MI as patients with coronary artery disease [13,14]. Individuals with early-stage PAD either do not experience or frequently under-report claudication symptoms (pain in the lower extremities) and despite the high in-hospital costs associated with advanced stage PAD, the disease often remains undetected and untreated [15,16,17].

A recent systematic review has revealed an estimated increase in PAD prevalence of more than 17% (30 million people) over a period of 5 years (2010–2015) from previous estimates of 202.06 million people suffering from PAD globally. In 2015, around 236 million people were estimated to have PAD globally, with a slightly higher percentage of women affected [18,19]. Regardless of advances in treatment modalities, outcomes have remained suboptimal not only in low- and middle-income countries but in countries with higher socioeconomic status as well, especially in patients with critical limb ischaemia (CLI) [20,21]. The number of PAD patients have been markedly increasing, resulting in an increase of related disease burden upon healthcare systems worldwide [22,23,24].

Symptomatic PAD is associated with marked changes in quality of life. PAD and diabetes are the major causes of lower limb amputations throughout the world [4,25].

Women are equally affected by the burden of PAD and frequently experience faster decline in quality of life and functional capacity than men, with minority women often having worse outcomes than white women [26]. Awareness of the disease is still strikingly low among at-risk populations and the general population as well [27,28,29,30]. Besides the significant reduction in quality of life, a considerable number of patients with PAD develop symptoms of depression. Depressive symptomatology among affected patients has been associated with limitations in physical functioning [31,32].

The treatment of PAD requires a multidisciplinary approach, addressing two primary aspects: specific symptoms and the risks and complications a specific lesion could potentially lead to, and reducing CV risk of these patients. Non-pharmacological measures are also crucial and may significantly contribute to reducing CV risk, including smoking cessation as the most important element lifestyle modification, together with a healthy diet and regular physical exercise. [10,33]. Supervised exercise programmes have been shown to be beneficial for patients with symptomatic PAD in terms of walking ability, functional status and HRQoL [34]. According to current guidelines, pharmacotherapy for PAD patients should include antiplatelet therapy and statin agents, taking into consideration additional risk factors such as diabetes mellitus or hypertension. Glycaemic control and antihypertensive therapy are vital in reducing the incidence of future CV events in symptomatic PAD patients. Choosing the optimal antithrombotic regimen to reduce ischaemic cardiac and limb events without increasing the risk of major and life-threatening bleeding has been a challenging and extensively studied issue given the heterogeneity of PAD patients [35,36].

A recent analysis of the EUCLID (Examining Use of Ticagrelor in Peripheral Artery Disease) trial showed increasing frequency of ischemic cerebrovascular events over time in this population, which underlines the importance of PAD as a potential risk factor of stroke and transient ischemic attack (TIA) [11]. The aim of our review is to provide an in-depth overview of the topic to gain up-to-date information about the prevalence, risk factors, consequences, and burden of this phenomenon a stroke physician should be aware of. We also discuss the situation of PAD management in Hungary, a country performing at the lower end of the spectrum with regard to prevention, treatment and amputation rates. 

We performed a narrative review. A literature search was conducted using the Medline Pubmed database with the following search terms: peripheral artery disease, epidemiology, prevalence, mortality and years of life lost. Two experts screened the abstracts and identified the most relevant papers. Primary data were extracted from the global burden of disease (GBD) study [2]. The GBD database was accessed through the internet from www.healthdata.org provided by the Institute for Health Metrics and Evaluation [37].

## 2. PAD Prevalence Worldwide

The 20th century witnessed dramatic advancements in the history of healthcare and medicine resulting in the so-called ‘epidemiologic transition’. Industrialisation and urbanisation resulted in a shift in major causes of morbidity and mortality, primarily in societies with more advanced socioeconomic status, from nutritional deficiencies and infectious diseases towards degenerative chronic diseases e.g., cardiovascular diseases, cancers and diabetes. The epidemiologic transition has not ended; different global regions, countries or even subgroups of a given country’s population are undergoing different stages of the process. As a consequence of the above phenomenon, from among noncommunicable diseases, atherosclerotic CVD has become a global epidemic accounting for alarmingly high mortality rates. Countries that have been successful in their efforts to prevent, diagnose, and treat cardio and cerebrovascular diseases have had to face the problems posed by an aging society upon their healthcare systems [3].

The epidemiology of PAD has been studied more extensively in Western countries since the last decade of the twentieth century, resulting in comprehensive descriptions of the disease, its aetiology, prevention and therapeutic modalities. The increasing global burden of CVDs and other NCDs in LMICs mandated epidemiological data to be updated and provide a clearer picture of the immensity of the problem societies and healthcare systems have to tackle [1,2,3]. Despite LMICs having been mostly impacted by the epidemiological transition, epidemiological studies from these countries are still scarce.

It was the Global Peripheral Artery Disease Study that first established the global and regional prevalence of PAD in the general population using the ankle-brachial index (ABI; the ratio of the systolic blood pressure at the ankle to the systolic blood pressure in the arm) of 0.90 or less as a diagnostic tool and estimated the prevalence of PAD to be around 202 million worldwide in 2010 with approximately 70% of the affected population living in LMICs [18]. 

An updated systematic review and analysis studied the prevalence of PAD in the general population at global, regional and national levels. ABI ≤ 0.9 was used as an indicator of disease. Age-specific and sex-specific prevalence of PAD was compared in high-income countries (HICs) and low-income and middle-income countries (LMICs). Authors used UN population data to generate the number of people suffering from PAD in 2015. According to the review, there was a slightly higher prevalence in LMICs than in HICs (4.32% vs. 3.54% at 40–44 years) among younger individuals; nevertheless, HICs witnessed greater increase with age, resulting in a higher prevalence in HICs than LMICs at older ages (21.24% vs. 12.04% at 80–84 years). Regarding sex differences, LMICs showed little difference between men and women (i.e., 6.40% vs. 6.37% at 55–59 years). The study estimated an overall, global prevalence of PAD in the population aged 25 years or above to be 5.56%, with a higher estimated prevalence in HICs than in LMICs (7.37% vs. 5.09%) which meant an estimated total number of 236.62 million people aged 25 years and older suffering from the disease in 2015 worldwide, with 72.91% living in LMICs. Women were slightly more affected, accounting for 52.23% of the PAD population. Authors identified smoking, diabetes, hypertension, and hypercholesterolaemia as major risk factors of PAD. Among all WHO regions, the Western Pacific Region (WPR) had the highest number of PAD cases (74.08 million), and the Eastern Mediterranean Region (EMR) was found to have the lowest case numbers (14.67 million) globally. The prevalence of PAD was the highest in the European Region (7.99%) with the age group 45–54 years being mostly responsible for high case numbers. Prevalence was the lowest in the African region (4.06%). Fifteen countries had case numbers accounting for more than two thirds (68%) of the global prevalence, with China, India and the USA having reported the highest case numbers [19]. 

Studies from the USA revealed high mean, annualised prevalence of PAD (10.69%) and CLI (1.33%) among Medicare and Medicaid beneficiaries [38]. Higher age-standardised incidence and prevalence was found in the black population especially among black women, compared with the white population. A recent systematic review and meta-analysis also highlighted the nearly twice as high mean prevalence of PAD among the general population for black people (6.7%) compared to white and Asian populations (3.5% and 3.7% respectively), emphasizing existing racial disparities within the American population and the need for more comprehensive and extensive prevention programmes [39]. Another systematic review also found higher PAD rates among women in general compared to men (3.8% vs. 3.2%), especially in the diabetic population in the USA (13.7% vs. 10%) [40]. 

Studies from Europe have also revealed a rapid increase in the prevalence of PAD in the European region. From previous estimates of 5.3% of the population living with the disease in 2010, which meant 40 million individuals living with PAD from among the 750 million inhabitants a decade ago, a later study gave estimates of around 50 million having PAD in the European region in 2015, with around 33 million living in HICs [18,19]. The PANDORA study including 10,287 patients highlighted significant variations in PAD prevalence in Europe, with Greece having the highest prevalence (28.0%), followed by Italy (22.9%), France (12.2%), Switzerland (12.2%), the Netherlands (8.1%) and Belgium (7.0%) [41,42].

Early studies from Northern Europe established the high prevalence of PAD among the elderly (19.1% in the Netherlands, 18% in Sweden) and called attention to the fact that the vast majority of PAD patients remain asymptomatic [6,12,20]. Asymptomatic PAD and CLI were more prevalent among women (12.6% vs. 9.4%; 1.5% vs. 0.8% respectively) [20]. Incidence and prevalence were found to be strongly affected by age and PAD prevalence was higher among women. Asymptomatic PAD was found to be associated with age, smoking status, hypertension and diabetes. CLI was found in around 1% of study populations [6,14,20]. 

In 2019, according to Global Burden of Disease (GBD) data retrieved from vizhub.healthdata.org [37], the global prevalence of PAD per 100,000 population was 332.32 in males and 621.11 in females in the 40–44 age group and increased markedly with age, resulting in prevalence rates of 17,195.57 in males and 24,965.3 in females aged 95 and older.

**Figure 1:**
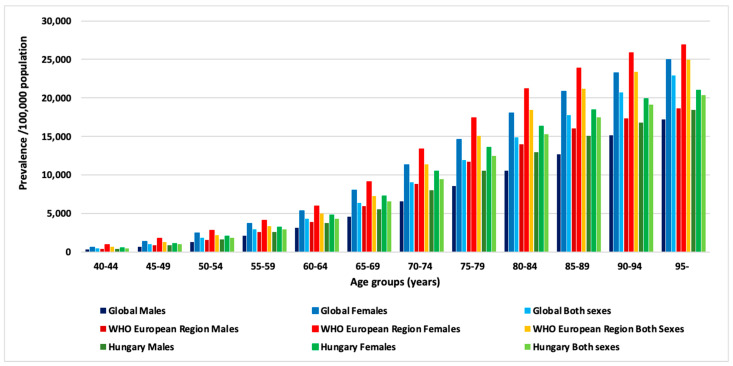
Prevalence of PAD per 100,000 population by age group in males, females and both sexes combined, in 2019. (Graph by authors, source: https://vizhub.healthdata.org, access date: 28 March 2022).

The WHO European region showed similar rates with a prevalence of 407.1 among males and 940 among females aged 40–44, and significantly increased rates of 18,664.74 in men and 26,896.52 in women aged 95 years and above. As for Hungary, in 2019, PAD was less prevalent among males than among females with a rate of 443.83 at the age 40–44 years increasing up to 18,448.15 by age 95. In women, prevalence rates were higher compared to the above rates with 529.86 in the 40–44 age group, and 20,962 in women aged 95 and older. Females showed overall higher prevalence rates in all age groups, which corresponds with findings of earlier studies and a need for action targeting the female population. Generally, PAD prevalence shows marked increase with age especially above age 65 years (Figure 1). 

## 3. Mortality Attributed to PAD

It is often argued that mortality statistics are of limited usefulness with respect to PAD, as the majority of these patients die from coronary heart disease, stroke or cancer and thus the cause of death is identified as such. A very small minority die of PAD only and consequently mortality statistics may not provide clear insights in this respect [43]. 

A systematic review analysing randomised and observational studies between 2003–2017 revealed pooled event rates for all-cause and CVD mortality, MI, stroke, MACE, and major amputation as 113, 39, 20, 12, 71 and 70 per 1000 person-years, respectively, among PAD patients. Patients with CLI were identified to be at the highest risk of PAD-associated morbidity and mortality [13]. 

Based on data from the Global Burden of Diseases 2010 study, in 1990, the age-specific death rate per 100,000 population from PAD ranged from 0.05 in the 40–44 age group to 16.63 in people aged above 80 years worldwide. In 2010, estimates for the same age groups were 0.07 and 28.71, reflecting an increase in death rates related to PAD. Pad-related mortality increased considerably with age. 

Comparing Eastern and Western Europe, death rates attributed to PAD were found to increase between 1990 and 2010 from 1.37 to 4.58 per 100,000 population among women in Western Europe, and from 0.08 to 0.35 in Eastern Europe. Among men, changes in death rate attributable to PAD ranged from 1.37 to 2.00 in 1990 and 2010 in Western Europe and from 0.04 to 0.09 per 100,000 population for the same years in Eastern Europe [1].

**Figure 2:**
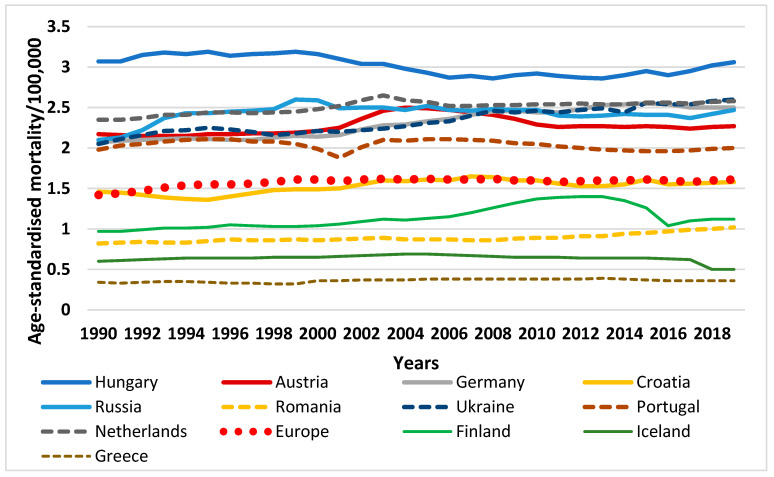
Age-standardised mortality due to PAD per 100,000 population in some European countries and Europe, among females, between 1990–2019. (Graph by author, source: https://vizhub.healthdata.org, access date: 28 March 2022).

**Figure 3:**
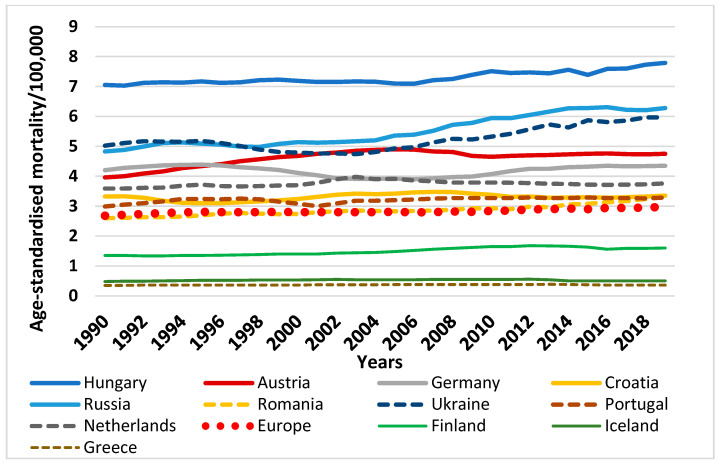
Age-standardised mortality due to PAD per 100,000 population in some European countries and Europe, among males, between 1990–2019. (Graph by authors, source: https://vizhub.healthdata.org, access date: 28 March 2022).

Taking a closer look at European countries, according to the most recent data from the GBD study, age-standardised mortality due to PAD per 100,000 population was considerably low in Greece in 1990 (0.35 in males, 0.34 in females), Iceland (0.48 in males, 0.6 in females) and Finland (1.35 in males, 0.97 in females) compared with the European average of 2.68 in males and 1.42 in females, with very small changes by the year 2019: Greece (0.36 in both sexes), Iceland (0.5 in both sexes) and Finland (1.6 in males, 1.12 in females) compared to the European average for 2019 of 2.98 deaths per 100,000 attributable to PAD. In contrast, Hungary had the highest death rates due to PAD in both sexes (7.05 in males, 3.07 in females) in 1990, and sadly maintained the highest rates in 2019 (7.79 in males, 3.06 in females), followed by Ukraine (5.02 in males), Russia (4.83 in males), and in the case of females, the Netherlands (2.35), Austria (2.17) and Russia (2.13) in 1990. The lowest mortality rates in 1990, in males, were found in Greece (0.35), Iceland (0.48) and Finland (1.35); in females, in Greece (0.34), Iceland (0.6) and Romania (0.82). In 2019, the same countries maintained top positions, following Hungary, in terms of PAD-related mortality with considerable increase among males in Ukraine (5.97) and Russia (6.28) and fewer changes among females in the Netherlands (2.58), Austria (2.58) and Russia (2.47). Countries with the lowest mortality rates experienced increases at significantly smaller scales: mortality rates in Greece rose to 0.36, in Iceland to 0.5, and in Finland to 1.6 among men. Among women, Iceland witnessed a slight decrease down to 0.5 deaths per 100,000. Greece had a slight increase up to 0.36 and Romania reported 1.02 deaths (Figure 2 and Figure 3).

According to GBD data, in 2019, PAD accounted for 0.13% of total deaths (0.074–0.23%) globally, including both sexes and all ages with a mean annual percentage change of 1.82% between 1990–2019 [37].

According to the same source, in 2019, PAD accounted for 0.78% (0.32–1.6%) of total deaths in Hungary. Among males, PAD accounted for 0.92% of total deaths (0.31–2.27%) with a mean annual percentage change of 1.81% between 1990–2019. Among females, PAD was responsible for 0.65% of the total death rate (0.24–1.65%); the mean annual percentage change was 2.04% during the same time period. Mean annual change in PAD mortality between 1990 and 2019 was 1.9% for both sexes in all ages. In 2019, 10.45 deaths per 100,000 (4.02–22.35) occurred due to PAD in Hungary [37]. 

## 4. PAD, Risk Factors, Comorbidities and Major Adverse Limb Events

Atherosclerosis is a systemic disease and around 60% of PAD patients are expected to have ischaemic heart disease and one third cerebrovascular disease [44]. Approximately 5 years after diagnosis, 10–15% of patients with IC are highly likely to die of CVD. Therefore, it is of pivotal importance to identify and target risk factors that are in the background of PAD, heart disease and stroke [45]. A systematic review including 20,278 patients from 17 studies concluded that around half of all PAD is attributable to smoking. Despite quitting, past smokers have a persistently increased risk compared with never smokers [46]. Diabetes is the other crucial modifiable risk factor. TASC II. guidelines stated that diabetes puts patients at equal risk of developing PAD as smoking [44]. Studies mentioned above also found that patients with ABI < 0.90 were more likely to be smokers, to have hypertension and to suffer from symptomatic or asymptomatic CVD. Marital and socioeconomic factors, especially in men, with separation/divorce, unemployment, and lower educational achievement, have been associated with a lower ABI [41,42]. Besides age and female sex, several smaller studies have also highlighted smoking and diabetes as important risk factors for the development of PAD.

Although authors found lower PAD prevalence in an urban population in South India compared to studies in Europe or the USA in 2000, PAD prevalence was 6.3% among diabetics [47]. Another study by Krishan et al. revealed high age-adjusted prevalence of PAD (26.7%) in Kerala, India. Asymptomatic PAD was more prevalent among women (25.35% vs. 20.37%, *p* = 0.0485). Authors attributed the high prevalence of PAD to the high frequency of risk factors, especially smoking [48]. 

The impact of ethnic background on PAD prevalence has also been extensively studied. According to a recent study, Cuban Americans were significantly afflicted by PAD (9.1%) and had a three-fold higher prevalence (OR: 2.9, 95% CI: 1.9–4.4) compared to Mexican Americans or other ethnic groups with Hispanic/Latino background. Authors suggested high smoking rate as a possible confounding factor besides genetic predisposition [49]. 

A high prevalence (15.0%) of PAD was revealed among the elderly (>65 years) in the general population of two cities in Central Africa; PAD prevalence showed an association with hypertension, diabetes and increasing age [50].

## 5. PAD, Diabetes and Major Adverse Limb Events 

Lower limb amputations are one of the most devastating consequences of PAD and diabetes [43]. Diabetic patients were shown to have a 5-fold higher amputation risk, worse PAD outcomes and higher mortality rates compared to non-diabetic patients with a similar history of smoking, ischaemic heart disease and hypercholesterolaemia in the UK. Even upon receiving up-to-date therapies, patients with PAD and diabetes are at higher risk of developing adverse limb events compared to those patients with PAD alone [51]. 

Outcomes of the COMPASS trial revealed that subsequent to the first major adverse limb event, the cumulative risk of repeated hospitalisation was 61.5%, for vascular amputations 20.5% and for death 8.3% among patients with LEAD. MALE was associated with significantly worse prognosis [21].

Race and socioeconomic status have been shown to independently affect the risk of a major amputation in PAD with black patients having a 37% higher risk than white patients. Black patients have been found to have more severe PAD status at presentation [52]. Studies have also shown that there has been no change with respect to racial disparities in vascular outcomes, as black patients continue to experience higher major adverse limb events (HR 1.15 [1.06–1.25], *p* < 0.001), and amputation rates (HR 1.33 [1.18–1.51], *p* < 0.001), irrespective of the region in the USA [53,54]. 

Caring for amputees poses a substantial economic burden for healthcare systems and results in a significant decrease in quality of life for the individual. Lower limb amputation rates as distal outcome indicators may shed light upon the effectiveness of various preventive and therapeutic approaches and thus may prove essential in evaluating vascular care [55].

## 6. Lack of PAD Awareness

Disease awareness is pivotal in designing and implementing targeted awareness campaigns. Despite the high prevalence and associated mortality risk of PAD, large population-based studies have demonstrated suboptimal levels of knowledge of PAD in the Netherlands (20% aware), Canada (36% aware), the United States (26% aware) and Ireland (18% aware) in the general population, and affected patient populations alike. Authors observed an unexpectedly low familiarity with PAD terminology, symptoms, and accompanying risk factors. Strikingly, familiarity with preventive and treatment options by lifestyle changes including cessation of smoking was also surprisingly low. Lower levels of education and lower socioeconomic status have been found to be associated with more significant gaps in public knowledge regarding risk factors, symptoms and consequences of PAD [27,28,29,30].

## 7. PAD and Depression

Functional impairment and pain resulting from lower extremity arterial ischaemia may lead to significantly reduced quality of life and, consequently, PAD patients may experience severe depressive symptoms. 

In a cross-sectional study comprising 300 individuals recruited from general practitioners’ offices, Tóth-Vajna et al. examined the association between depressive symptomatology and peripheral artery disease. The study revealed a strong relationship between depression and PAD with 63% prevalence of depression among ‘PAD-positive’ patients and 59% in symptomatic patients without ABI abnormalities; prevalence was 20% in the non-compressible artery group compared to 8% prevalence among those without any sign of PAD, the group the authors called ’clear PAD negative’ [31]. 

A study conducted in the United States also highlighted the increased prevalence of depressive symptoms among people suffering from PAD, mainly resulting from impaired lower extremity functioning [32]. 

## 8. Disease Burden: Direct and Indirect Costs of PAD

A comprehensive assessment of the global and regional burden of death and disability from PAD revealed that HICs are more affected by PAD than LMICs. Authors used the Disability Adjusted Life Years (DALY) to measure population burden and found the largest DALY rates, per 100,000 population, in the years 1990 and 2010, in the same high-income regions of Australasia, Western Europe, and North America. Regarding Europe, Central European countries were found to have DALY rates around half that of high-income Western European countries and twice as high as low-income Eastern European countries [1]. 

A recently published systematic analysis for the GBD Study 2016 investigated global, regional and national incidence, prevalence and years lived with disability (YLDs) for 328 diseases and injuries for 195 countries and territories from 1990 to 2016. PAD accounted for 520,000 (244,000–941,000) YLDs in 2016. Percentage change in counts between 2006 and 2016 was 25.5% (23.8–27.3); percentage change in age-standardised rates between 2006 and 2016 was −5.9 (−6.8 to −5.0). For the year 2016, authors revealed a less than two-fold difference in age-standardised YLD rates for all causes between China, the country with the lowest rate (9201 YLDs per 100,000; 6862–11,943 per 100,000) and Yemen, the country with the highest rate (14,774 YLDs per 100,000; 11,018–19,228 per 100,000). The relative contribution of YLDs to the total burden of disease in DALYs increased from 21.7% (17.2–26.6) in 1990 to 33.5% (27.4–39.7) in 2016, reflecting the fact that the aging of populations is a global phenomenon with the inevitable consequence of people have to live more years with diseases than before and the growing number of people needing chronic care [2].

**Figure 4:**
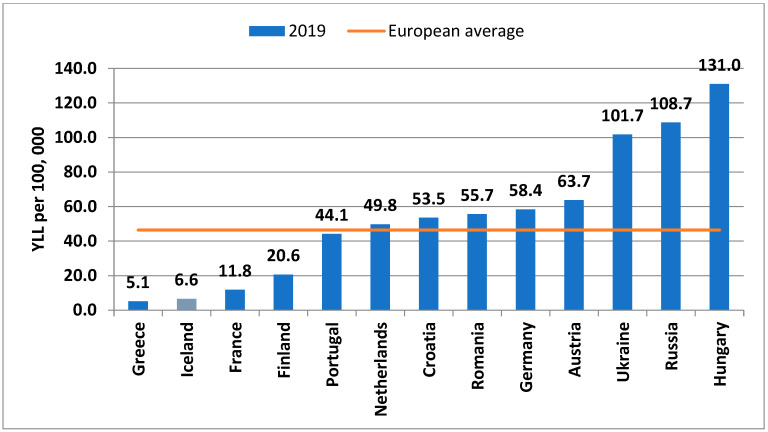
Age-standardised YLL per 100,000 in some European countries among men, in 2019 (Graph by authors, source: https://vizhub.healthdata.org, access date: 28 March 2022).

**Figure 5:**
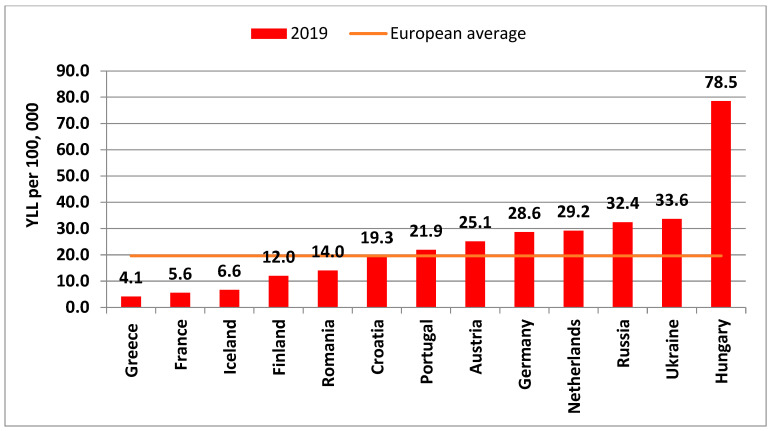
Age-standardised YLL per 100,000 in some European countries among women, in 2019 (Graph by author, source: https://vizhub.healthdata.org, access date: 28 March 2022).

Regarding Years of Life Lost (YLL), recent data from the Global Burden of Disease Study revealed alarmingly high rates of age-standardised YLL in Hungary in both sexes (131.0 in males, 78.5 in women per 100,000 population) in Europe, followed by Russia (108.7 in males, 32.4 in females per 100,000 population) and Ukraine (101.7 in males, 33.6 in females per 100,000 population) in 2019. Countries with the lowest YLL, per 100,000 population, in Europe in 2019 included Greece (5.1 in males, 4.1 in females), France (11.8 in males, 5.6 in females), Iceland (6.6 in both males and females) and Finland (20.6 in males, 12.0 in females) (Figure 4 and Figure 5).

## 9. Hungary

The present paper intends to devote special attention to Hungary, as our country has continued to have significantly high amputation rates, amputation-related mortality and PAD-related mortality. In Hungary, cardiovascular diseases account for more than half of total mortality [56]. 

The largest national epidemiological study with the inclusion of 21,892 men and women with hypertension (mean age 61.45 years) revealed very high prevalence of asymptomatic PAD (14.4%) in Hungary. Patients with ABI 0.91–0.99 accounted for 15.6% and patients with ABI > 1.3 accounted for 9.4% of the study population [57]. 

A retrospective cohort study was conducted within the framework of the HUNgarian VASCular DATA (HUNVASCDATA) project, based on healthcare administrative data for the whole Hungarian population covering the years 2004–2012. Authors analysed changes in PAD-related major amputation rates and revealed that there was no significant change in this respect in Hungary. Compared to the European Standard Population (ESP), the incidence of lower limb major amputations was drastically high at 42.3/100,000 in the total population, with 50.4% of amputees being diabetic. During the study period of 9 years, 76,798 lower limb amputations were performed, out of which 71.5% were primary amputations [58].

Started in 1997, VASCUNET is a collaboration of registries for vascular surgery in Europe, Australia, New Zealand, and Brazil with the aim to create a common international dataset on vascular surgery, to promote the understanding of vascular disease and share knowledge in the field of vascular surgery [59]. According to the 2018 VASCUNET report based on data reported by 12 countries including 259 million inhabitants, covering the period 2010–2014, Hungary had an alarmingly high incidence rate of major (above the ankle) amputations. In the ≥65 age group, Hungary had the highest rate of major amputations compared to the general population. The incidence of major amputations was 3.5 times higher among the elderly compared with all age groups. With an incidence of 41.4/100,000, Hungary performed at the highest end of the spectrum with the highest amputation-related mortality rate of 20.3%. Regarding smoking as a predisposing factor, the proportion of active smokers was also the highest in Hungary with 25.8%. Authors found an association between higher amputation rates, lower socioeconomic status and healthcare expenditures [60].

A more recent study investigating lower limb amputation and revascularisation procedures, highlighted a 10-year delay regarding the start of a declining tendency in amputation rates. The study by Kolossváry et al. analysed inpatient administrative data claims for the entire beneficiary population of Hungary over a period of 14 years (2004–2017) and included both major and minor amputations. Authors observed a slight decline in major amputations (15%) from 2013 and a 79% growth in the number of endovascular procedures performed (based on crude rates), a pattern observable in developed countries. As a consequence of ’endovascular first’ strategies being adopted and becoming increasingly widespread, other Western European countries witnessed this trend a decade earlier. In Hungary, the crossing of the two trend curves occurred in 2015. Despite the promising trend, authors emphasised the fact that compared to developing countries, endovascular procedures accounted for a significantly lower segment of the volume of vascular procedures during the period under investigation [55]. 

Early recognition of LEAD is of pivotal importance as patients with vascular diseases have 2–4-times more risk of developing cardio- and cerebrovascular events in the future [4,10]. The majority of PAD patients are asymptomatic, and thus may remain undiscovered and untreated without targeted screening [10,15]. A large epidemiological study with the inclusion of 816 individuals investigated the prevalence of PAD among patients screened in primary healthcare settings in Hungary with the aim to improve the efficacy of screening, with a special focus on patients with ABI negative symptomatic status or non-compressible arteries. Among the study population, 52% (*n* = 425) of the patients were clear PAD negative, 23% (*n* = 185) were clear PAD positive, 13% (*n* = 109) had normal ABI but were experiencing symptoms of walking impairment, and 12% (*n* = 97) belonged to the non-compressible artery group (ABI> 1.4). The risk factors profile of the ABI negative symptomatic subgroup was very similar to those of the other subgroups with hypertension (81%), diabetes (35%), active smoking (28%), myocardial infarction (23%) and stroke (13%) in the past medical history. In line with findings of the PAOD (Peripheral Artery Occlusive Disease) study [12], the above Hungarian study calls special attention to the high prevalence of PAD in the general population and the significant role of multiple risk factors, emphasizing the important role of general practitioners in the early recognition and treatment of PAD and stressing the importance of further testing in ABI negative patients [61]. 

Another study, also in a primary care setting in Northern Hungary, between 2015–2017, involving 680 patients, further emphasised the fact that among patients comprising the so-called ’murky zone’, i.e., ABI negative symptomatic patients (14%), and those with non-compressible arteries (12%), ABI screening may often not suffice in detecting patients suffering from PAD [62].

Apart from highlighting the high national prevalence of PAD and significant presence of risk factors, recent studies conducted in Hungary stress the importance of targeted screening for the disease, and the important role of general practitioners in preventive and therapeutic efforts especially in areas of the country with lower socio-economic conditions and suboptimal access to healthcare [61,62].

## 10. Conclusions

Considering an increasing life expectancy, and the global epidemiological transition towards an older age distribution, people are expected to live longer years with chronic conditions that significantly impact quality of life [3]. Chronic ill health causes a significant burden for patients, not only in terms of acute or chronic pain but also as a result of limitations that may alter patients’ ability to work, to engage in social activities, or to be actively involved in family life. The global burden of PAD has increased over the past decades with developing regions of the world having witnessed a more striking increase in PAD-attributable disease burden [1,2]. Administrative data may not be sensitive enough for identifying all forms of PAD in communities [17]. 

Consequently, early detection of PAD, especially among asymptomatic patients or those with atypical symptoms, is of pivotal importance as PAD has remained underdiagnosed and undertreated [14,17,61,62].

Regardless of advances in treatment modalities, outcomes especially among patients with CLI have remained unfavourable in LMICs and HICs as well [21]. The number of PAD patients have been markedly rising, causing an increasing disease burden to healthcare systems globally [1,2]. PAD is associated with significant morbidity and mortality from cardio- and cerebrovascular diseases and patients have an equal risk of suffering a future stroke or MI as patients with coronary artery disease [13].

The management of PAD patients accounted for considerably high percentages of in-hospital and healthcare costs [14]. PAD is associated with considerable physical and psychosocial disease burden primarily, due to impaired functional status and deterioration in quality of life [22,23,25].

Despite the spread of up-to-date limb salvage interventions, the number of PAD-related major and minor amputations has not decreased at optimal rates, especially in Eastern European countries [55,60].

A comprehensive knowledge of the disease burden attributable to PAD, the evaluation of the improvement in quality of life achieved via the latest pharmacotherapeutic and surgical interventions, together with addressing the issues relating to risk factor reduction, particularly the promotion of smoking cessation and the management of diabetes mellitus and hypertension are, therefore, equally important in both developed and developing regions of the world [63]. The management of CV risk factors includes smoking cessation, healthy diet, physical activity, and controlled exercise training. Pharmacological therapy is aimed at the management of hypertension, diabetes, lipid control (statin, ezetimibe) and adequate antiplatelet therapy [10]. Resource-limited countries will also need to prioritise early detection and treatment of PAD as this disease is expected to remain a major and crucial public health challenge in the foreseeable future.

## Data Availability

Not applicable.

## Abbreviations

CVD	cardiovascular disease
LMICs	low-income and middle-income countries
PAD	peripheral artery disease
GBD	global burden of disease
YLL	years of life lost
NCDs	noncommunicable diseases
LEAD	lower extremity peripheral artery disease
CV	cardiovascular
APAD	asymptomatic peripheral artery disease
MI	myocardial infarction
MACE	major adverse coronary events
CLI	critical limb ischaemia
ABI	ankle-brachial index
HICs	high-income countries
WHO	World Health Organisation
WPR	Western Pacific Region
EPR	Eastern Pacific Region
IC	intermittent claudication
DALY	disease adjusted life years
YLDs	years lived with disability
ESP	European standard population
